# Clinical and laboratory characteristics of hidradenitis suppurativa in a Chinese cohort: a retrospective analysis of 197 cases

**DOI:** 10.3389/fmed.2025.1721105

**Published:** 2026-01-12

**Authors:** Qian Ye, Chong Zhang, Xin Tang, Yan Yang, Yi Li, Yan Yan, Baoxi Wang

**Affiliations:** Department of Dermatology, Plastic Surgery Hospital, Chinese Academy of Medical Sciences and Peking Union Medical College, Beijing, China

**Keywords:** acne inversa, hidradenitis suppurativa, clinical characteristics, laboratory features, disease severity

## Abstract

**Background:**

The clinical and laboratory characteristics of hidradenitis suppurativa (HS), particularly indicators related to disease severity, remain inadequately explored in Asian populations.

**Objectives:**

To characterize the clinical and laboratory features of HS in Chinese patients and to identify risk factors for disease severity.

**Methods:**

We retrospectively analyzed 197 patients with HS in China. Demographic, clinical, and laboratory data were collected. Disease severity was graded using the Hurley staging system. Comprehensive statistical analyses were conducted.

**Results:**

Among 197 patients, 87.8% were male (male-to-female ratio 7.2:1). Females had a shorter diagnostic delay than males (*p* = 0.011). Obesity was present in 33.0% of HS patients. Metabolic abnormalities included reduced HDL-c (30.0%), elevated blood glucose (29.1%), increased TG (14.93%), TC (10.31%), and LDL-c (8.96%). Neutrophil-related indices (WBC, ANC, NEUT%) and liver function markers (TP, albumin, ALT, AST, TB) were significantly associated with progression to Hurley stage III. ROC analysis showed modest discrimination for these indicators (AUCs, 0.605–0.652). In multivariable logistic regression analysis, TP remained an independent risk factor for progression to Hurley stage III (OR, 1.240; 95% CI, 1.101–1.397; *p* = 0.010). A multivariable logistic regression model that integrated significant predictors achieved an AUC of 0.689. This indicated moderate discrimination for advanced disease severity.

**Conclusion:**

Chinese patients with HS exhibited a pronounced male predominance and relatively mild metabolic abnormalities. Neutrophil-related indices and TP, especially when TP was interpreted together with albumin, were associated with progression to Hurley stage III.

## 1 Introduction

Hidradenitis suppurativa (HS), also known as acne inversa (AI), is a chronic inflammatory skin disease. It is characterized by recurrent abscesses, draining tunnels, and hypertrophic scars that predominantly affect the intertriginous region. The global prevalence of HS is ~1%, varying by regions and populations (1). Beyond the skin, HS is associated with systemic inflammation and metabolic comorbidities (1).

The pathogenesis of HS is multifactorial. It has been reported to involve follicular occlusion, immune dysregulation, and cytokine-driven inflammation. Key contributors include IL-1β, IL-6, IL-17, IL-23, and TNF-α, while genetic predisposition and microbial dysbiosis further sustain immune cell infiltrates and tissue destruction (2). Mutations in γ-secretase-related genes such as *NCSTN* and *PSENEN* have been described in familial HS (3). Variants in immune-regulatory genes (e.g., *DEFB104B, GRAMD4*), (4) cytochrome P450 pathway genes, (5) and keratinocyte signaling pathways have been implicated in disease chronicity, heterogeneity in clinical course, and differential treatment responses (6, 7).

Epigenetic dysregulation also plays an important role in HS pathogenesis. Recent studies have identified aberrant DNA methylation of cytokine and chemokine genes (e.g., *CXCL10, CXCR6*), hypermethylation of immune-regulatory pathways such as interferon-γ, JAK-STAT, and IL-17 signaling, as well as disrupted hydroxymethylation and histone acetylation. Together, these alterations may drive chronic inflammation, impaired wound healing, and contribute to systemic immune dysfunction and increased cancer susceptibility in HS (4, 8). In addition, dysregulated expression of genes related to glucose metabolism *(POMC, IRS1, GNAS, CACNA1C, AHR, NOTCH3*), transporters (*ABCC2, ABCG1, SLC39A8, SLC39A9*), and metal homeostasis *(MMP2, MMP3, SOD2, CP*) may promote energy deficiency, ion imbalance, and oxidative stress, thereby amplifying inflammation, enhancing pain sensitization, hindering tissue repair, and ultimately facilitating tunnel formation and fibrosis (9).

The Hurley staging system remains the most widely used tool for assessing HS severity and guiding treatment. However, its reliance on anatomical features limits the ability to capture disease activity and progression, particularly in distinguishing between Hurley stages II and III (6). Therefore, identifying objective markers is crucial for improving disease assessment and management.

A broad range of systemic inflammatory markers has been investigated as potential indicators of HS severity. These include CRP, ESR, NLR, PLR, SII, PIV, SIRI, and SAA, and pro-inflammatory cytokines such as IL-6, IL-8, IL-12, and TNF-α (10–14). Autoantibodies against nuclear antigens (e.g., dsDNA, nucleolin, La/SSB), citrullinated proteins, and extracellular matrix components have also been implicated in HS pathogenesis (14). Serum proteins associated with neutrophil activity, including LCN2, G-CSF, and CXCR, show positive correlations with disease severity (15, 16). Additional biomarkers, such as RBP4, Ang-2, and ANGPTL2, have also been proposed as severity indicators (17). Dyslipidemia, characterized by elevated TG and reduced HDL levels, may also contribute to disease progression (18). However, inconsistent findings and small sample sizes limit their clinical applicability of these indicators, and evidence from Asian populations remains scarce.

To address this gap, we conducted a retrospective study to characterize the clinical and laboratory features of Chinese patients with HS and identified risk factors associated with progression to Hurley stage III, aiming to improve clinical stratification and management strategies.

## 2 Materials and Methods

This retrospective study analyzed the medical records of 223 Chinese HS patients who visited the outpatient dermatology clinic at the Plastic Surgery Hospital, Chinese Academy of Medical Sciences, between January 2016 and March 2025. All patients met the diagnostic criteria for HS according to established guidelines, which remained unchanged during the study period (19). Laboratory tests were performed under standardized protocols, and institutional quality assurance records confirmed the consistency of testing platforms and reference ranges over the study years.

Patients were excluded according to the following criteria: (a) receipt of systemic antibiotics or immunosuppressive therapy within the 4 weeks prior to consultation. (b) concurrent acute infections and/or (c) pregnancy or breastfeeding at the consultation. After applying these criteria, 197 patients were included in the final analysis.

Demographic and clinical variables included age, sex, body mass index (BMI), smoking status, age at disease onset, and family history. BMI was categorized according to World Health Organization (WHO) criteria: normal weight (18.5–24.9 kg/m^2^), overweight (25.0–29.9 kg/m^2^), and obesity (≥30.0 kg/m^2^). Laboratory investigations comprised complete blood count, liver and renal function tests, coagulation profile, and inflammatory markers such as C-reactive protein (CRP), interleukin-6 (IL-6), serum amyloid A (SAA), and procalcitonin (PCT). Because not all patients underwent every test, missing values were imputed using the mean for continuous variables and the mode for categorical variables, and pairwise deletion was applied in correlation analyses.

Descriptive statistics summarized demographic and clinical variables. Normality of continuous variables was assessed using the Shapiro–Wilk test. Normally distributed variables were analyzed using Student's *t*-test or one-way ANOVA, while non-normally distributed variables were evaluated using the Mann-Whitney *U* test or Kruskal-Wallis test. Categorical variables were compared using the chi-square test or Fisher's exact test.

Correlation analyses examined associations between laboratory markers and clinical indices. Pearson's correlation was applied for normally distributed data, and Spearman's rank correlation for non-normally distributed data. The correlation coefficient (*r*) was used to quantify the strength and direction of associations. For each correlation, 95% confidence intervals were calculated. Both raw and false discovery rate (FDR)-adjusted *p*-values (q value) were reported using the Benjamini–Hochberg procedure.

Logistic regression identified predictors of severe disease (Hurley stage III vs. Hurley stage I and II). Univariable logistic regression models were first fitted for each demographic, clinical, and laboratory variable. Variables with *q* < 0.05 in univariable analyses were further considered in multivariable logistic regression. To avoid multicollinearity, highly correlated predictors (*r* >0.7) were excluded prior to model fitting. Results are presented as odds ratios (ORs) with 95% confidence intervals (CIs), and corresponding raw and FDR-adjusted *p*-values (*q* value).

Model performance was evaluated using receiver operating characteristic (ROC) analysis. ROC curves and the area under the curve (AUC) values were generated for significant predictors (*q* < 0.05) from univariable analyses. An overall ROC curve with AUC was calculated for the multivariable model to evaluate combined predictive performance.

All analyses were performed using R software (version 4.4.2). Statistical significance was defined as *p* < 0.05 and/or FDR-adjusted *p* (*q* value) <0.05.

## 3 Results

### 3.1 Clinical characteristics

Among the 223 HS cases identified in our hospital information system, 197 patients were included in the final analysis, and their clinical characteristics are summarized in Table 1. Most patients were male (87.8%), with a male-to-female ratio of 7.21. The average age was 33.09 ± 10.36 years, with no significant age difference across Hurley stages (*p*_1_ = 0.12) or between genders (*p*_2_ = 0.82). Males had significantly greater weight and height than females (both *p*_2_ <0.001), while BMI did not differ by gender. Smoking was more common in Hurley stage III (64.1%) than in Hurley stage I (42.9%; *p*_1_ = 0.09), and was significantly higher in males than females (63.2 vs. 14.3%, *p*_2_ <0.001). A positive family history was reported in 24.7% of patients, with no significant differences across Hurley stages (*p*_1_ = 0.22) or genders (*p*_2_ = 0.32). The mean onset age of disease was 19.85 ± 5.84 years. Diagnostic delay increased with disease severity, with 6.25 ± 5.86 years in Hurley stage I, 8.62 ± 9.83 years in Hurley stage II, and 9.92 ± 9.03 years in Hurley stage III, although the differences were not statistically significant (*p* = 0.177). However, males experienced significantly longer diagnostic delays than females (9.42 ± 9.31 vs. 6.33 ± 4.57, *p* = 0.011). In our cohort, 12.6% of patients had Hurley stage I, 30.4% had stage II, and 57.1% had stage III, with no significant gender difference (*p*_2_ = 0.13). Notably, none of the patients had concomitant inflammatory bowel disease (IBD).

**Table 1:** Characteristics of 197 patients with hidradenitis suppurativa enrolled in the study.

**Characteristics**	**Hurley stage I (*n* = 24)**	**Hurley stage II (*n* = 58)**	**Hurley stage III (*n* = 109)**	***p*_1_**	**Male (*n* = 173)**	**Female (*n* = 24)**	***p*_2_**	**Total (*n* = 197)**
**Demographics**								
Age (years, mean ± SD)	29.83 ± 8.60			0.12	33.23 ± 10.68	32.04 ± 7.84	0.82	33.09 ± 10.36
**Gender**								
Male, *n* (%)	20 (83.3)	47 (81.0)	100 (91.7)	0.13	–	–	–	171 (87.8)
Female, *n* (%)	4 (16.7)	11 (19.0)	9 (8.3)		–	–	–	24 (12.2)
**Anthropometrics (mean** ±**SD)**								
Weight (kg)	84.7 ± 19.39	85.11 ± 22.26	89.21 ± 18.84	0.20	90.15 ± 19.62	72.05 ± 17.55	<0.001	87.02 ± 19.84
Height (m)	1.77 ± 0.08	1.76 ± 0.07	1.77 ± 0.08	0.37	1.78 ± 0.06	1.65 ± 0.05	<0.001	1.77 ± 0.08
BMI (kg/m^2^)	27.05 ± 5.38	27.39 ± 6.49	28.32 ± 5.04	0.28	28.25 ± 5.50	26.38 ± 6.01	0.32	28.03 ± 5.58
**Clinical features**								
**Smoking**, ***n*** **(%)**								
Positive	9 (42.9)	27 (50.0)	66 (64.1)	0.09	91 (63.2)	3 (14.3)	<0.001	104 (56.5)
Negtive	12 (57.1)	27 (50.0)	37 (35.9)		53 (36.8)	18 (85.7)		80 (44.5)
**Family history**, ***n*** **(%)**								
Positive	9 (42.9)	10 (18.5)	26 (25.7)	0.22	39 (27.1)	3 (14.3)	0.32	45 (24.7)
Negative	12 (57.1)	44 (81.5)	75 (74.3)		105 (72.9)	18 (85.7)		137 (75.3)
Age of onset (years)	19.83 ± 6.18	18.78 ± 4.76	20.85 ± 6.77	0.10	19.87 ± 5.87	21.29 ± 7.84	0.82	19.85 ± 5.84
Diagnostic delay (years, mean ± SD)	6.25 ± 5.86	8.62 ± 9.83	9.92 ± 9.03	0.177	9.42 ± 9.31	6.33 ± 4.57	0.011	9.04 ± 8.92
Hurley stage, *n* (%)	–	–	–	–			0.13	
Hurley stage I	–	–	–		20 (11.6)	4 (16.7)		24 (12.6)
Hurley stage II	–	–	–		47 (27.2)	11 (45.8)		58 (30.4)
Hurley stage III	–	–	–		100 (57.8)	9 (37.5)		109 (57.1)
**Inflammatory bowel disease**, ***n*** **(%)**								
Positive	–	–	–	–	–	–	–	0 (0)
Negtive	–	–	–	–	–	–	–	197 (100)

### 3.2 Metabolic characteristics

In our HS cohort, 58 (29.9%) had a normal BMI, 72 (37.1%) were overweight, and 64 (33.0%) were obese. Overall, 70.1% of patients were overweight or obese. Among metabolic parameters, abnormal HDL-c (30.0%) and blood glucose (29.1%) were most frequent, followed by TG (14.93%), TC (10.31%), and LDL-c (8.96%). TG levels were significantly higher in obese patients compared with both the normal-weight and overweight groups (*p* < 0.001). No significant BMI-related differences were found for glucose, TC, LDL-c, or HDL-c. Moreover, females showed higher HDL-c levels than males (*p* = 0.034), with no statistically significant differences in other metabolic indices. No significant differences in glucose or lipid parameters were found across Hurley stages I–III (Table 2).

**Table 2:** Comparison of metabolic indicators among patients with different groups.

**Variable**	**Blood glucose (mmol/L)**	**TC (mmol/L)**	**TG (mmol/L)**	**LDL-c (mmol/L)**	**HDL-c (mmol/L)**
Abnomal frequency (*n*%)	29.10%	10.31%	14.93%	8.96%	30.00%
**Gender**					
Male (*n* = 173, 87.8%)	5.60 (5.23–6.30)	4.43 ± 0.98	1.31 (0.94–1.80)	2.96 ± 0.83	0.99 (0.85–1.20)
Female (*n* = 24, 12.2%)	5.70 (5.27–6.35)	4.52 ± 0.92	1.35 (0.74–1.80)	3.02 ± 0.74	1.23 (1.09–1.46)
*p*-Value	0.918	0.756	0.510	0.765	0.034
**BMI**					
Normal BMI range, 18.5–24.9 (*n* = 58, 29.9%)	5.45 (5.20–5.80)	4.22 ± 1.04	0.95 (0.77–1.33)	2.90 ± 0.88	1.04 (0.87–1.26)
Overweight, 25.0–29.9 (*n* = 72, 37.1%)	5.60 (5.30–6.20)	4.50 ± 1.00	1.24 (0.95–1.69)	2.98 ± 0.80	1.10 (0.96–1.22)
Obesity, 30 or more (*n* = 64, 33.0%)	5.80 (5.30–7.60)	4.56 ± 0.91	1.68 (1.23–2.28)	3.06 ± 0.81	0.96 (0.85–1.14)
*p*-Value	0.067	0.327	<0.001	0.687	0.27
**Hurley stage**					
Hurley stage I (*n* = 24,12.6%)	5.80 (5.55–6.55)	4.34 ± 1.11	1.19 (0.83–2.21)	2.88 ± 0.92	1.11 (0.96–1.17)
Hurley stage II (*n* = 58, 30.4%)	5.40 (5.10–6.08)	4.42 ± 0.83	1.18 (0.89–1.69)	2.89 ± 0.73	1.16 (0.94–1.34)
Hurley stage III (*n* = 109, 57.1%)	5.60 (5.30–6.30)	4.49 ± 1.02	1.40 (0.96–1.81)	3.01 ± 0.85	0.99 (0.85–1.19)
*p*-Value	0.352	0.858	0.476	0.743	0.451

Values are presented as mean ± SD for normally distributed variables or median (Q1–Q3) for non-normally distributed variables.

TG, triglyceride; TC, total cholesterol.

### 3.3 Correlation analysis

**Figure 1:**
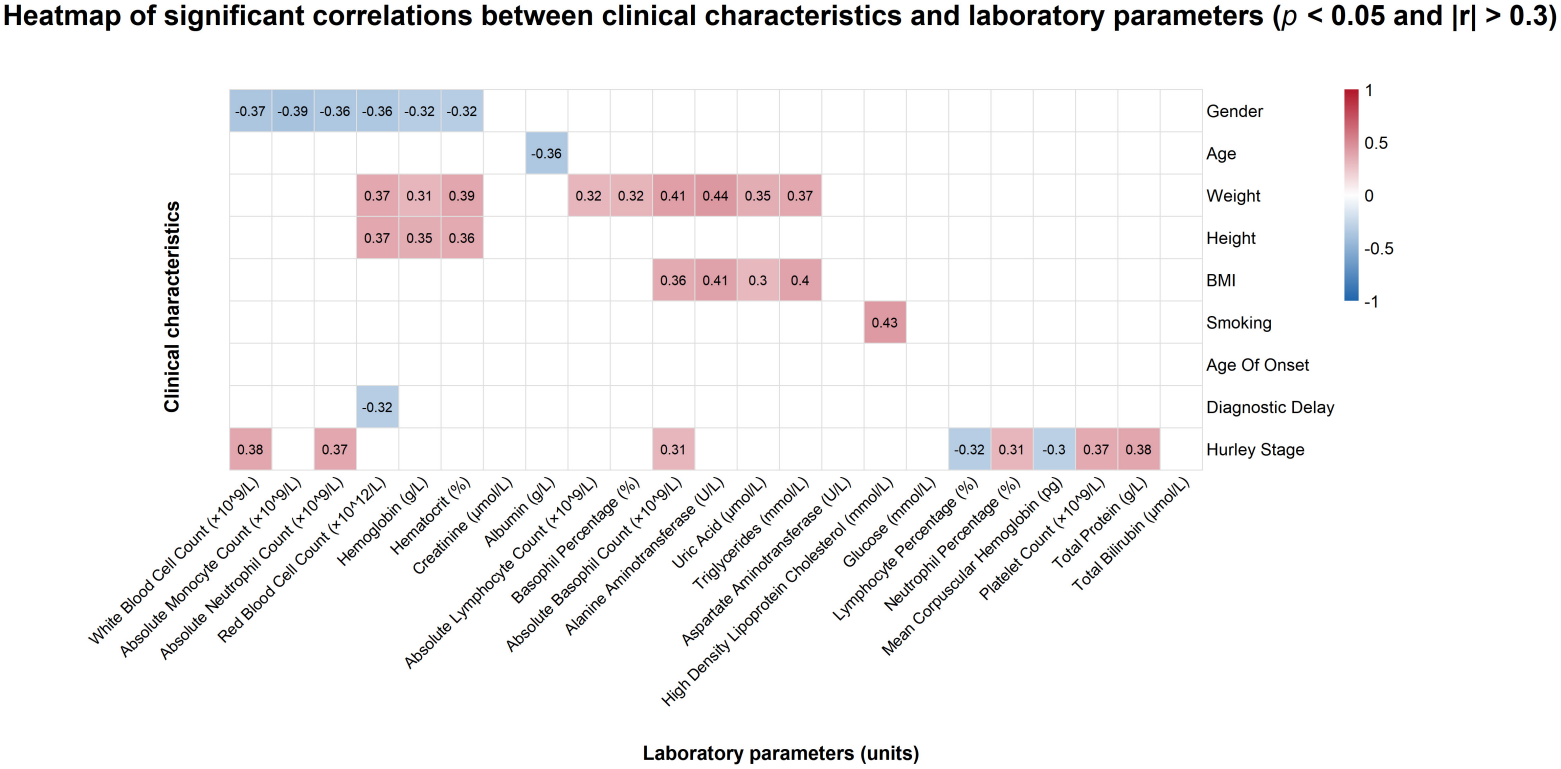
The correlation heatmap between clinical and laboratory variables displays only correlations with *p* < 0.05 and |*r*| > 0.3. Blue indicates a negative correlation, and red indicates a positive correlation.

**Figure 2:**
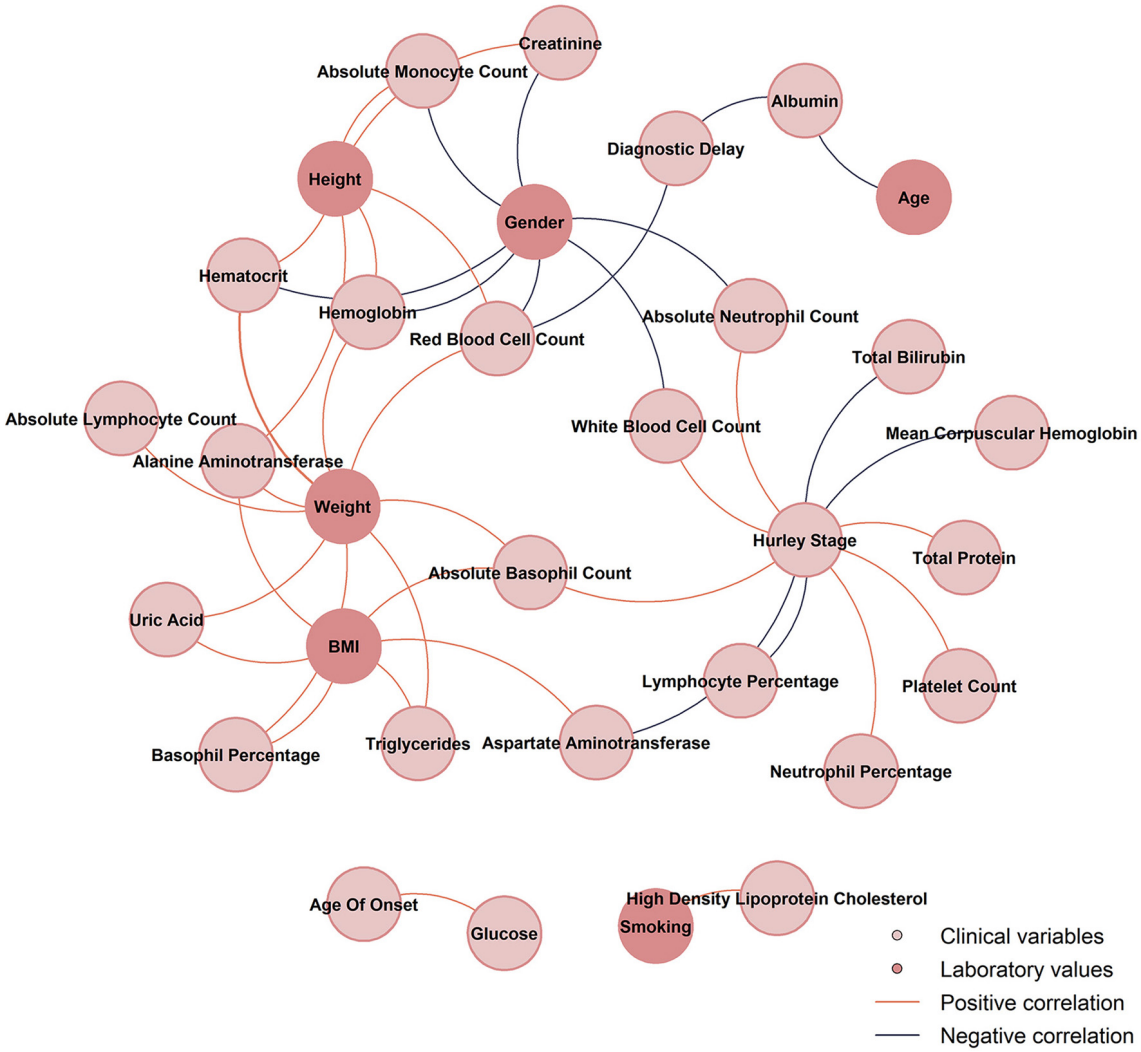
The network diagram between clinical and laboratory variables displays only correlations with *p* < 0.05 and |*r*| > 0.3.

Our correlation analysis revealed a wide range of significant correlations between clinical characteristics and laboratory parameters in HS. These results are detailed in Figures 1, 2 and Supplementary File 1. Males were positively associated with higher WBC, AMC, ANC, RBC, Hemoglobin, and Hematocrit compared with females. Body weight correlated significantly with RBC, Hemoglobin, Hematocrit, ALC, BAS%, ABC, ALT, UA, and TG. Moreover, BMI was positively correlated with ABC, ALT, UA, and TG. Notably, smoking was negatively associated with HDL-c.

Furthermore, WBC, ANC, NEUT%, ABC, PLT, and TP increased significantly with disease severity, whereas LY% and MCH showed significant inverse associations. Moreover, correlation analysis also demonstrated that the Hurley stage was positively associated with fibrinogen (*r* = 0.315, *p* = 0.035, *q* = 0.193) and CRP (*r* = 0.276, *p* = 0.133, *q* = 0.416), but negatively with albumin (*r* = −0.204, *p* = 0.019, *q* = 0.132).

### 3.4 Logistic regression analysis

**Figure 3:**
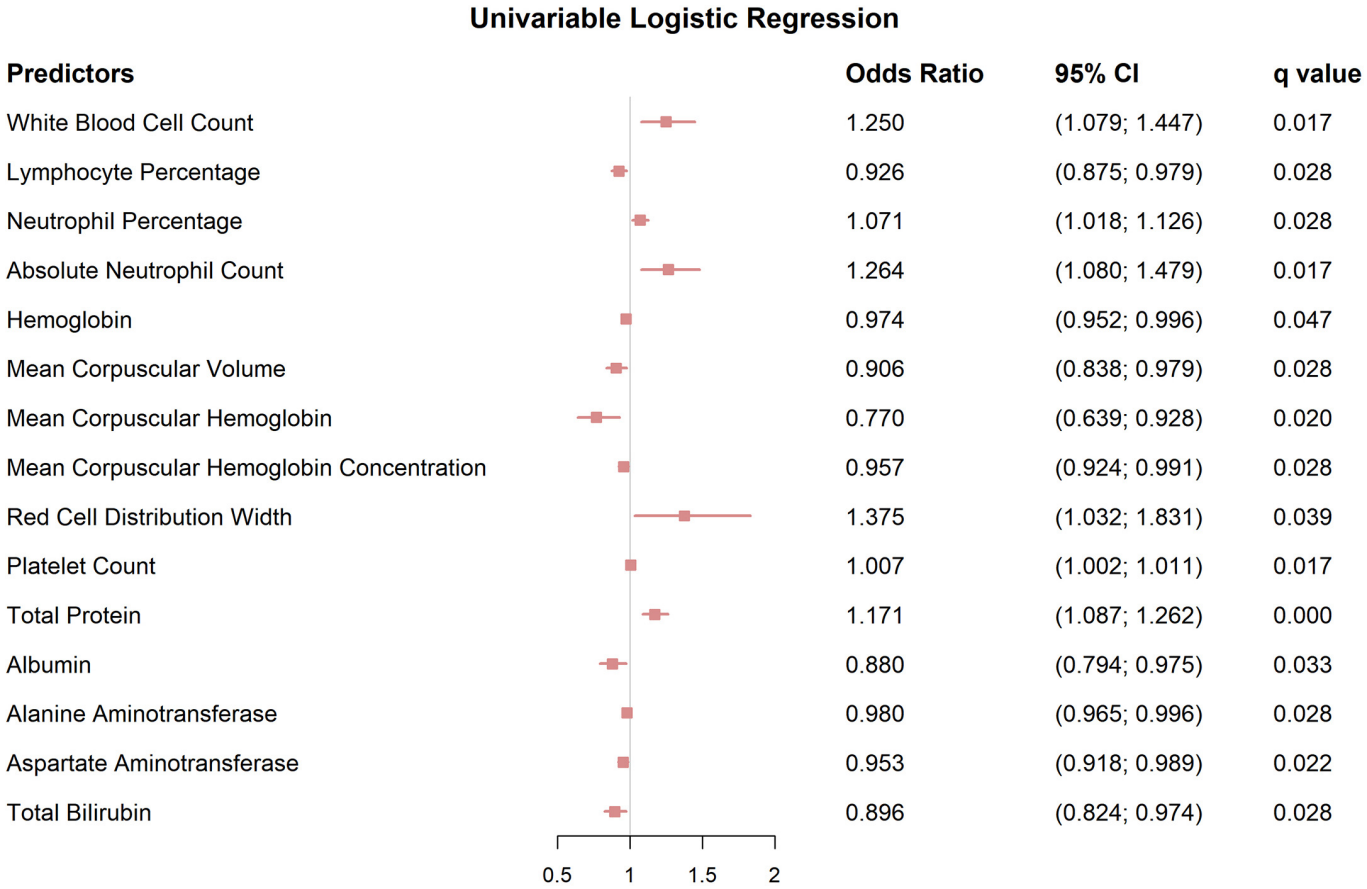
Forest plots of univariable logistic regression analyses identifying significant predictors of disease severity in HS, with *q* values, odds ratios (OR), and 95% confidence intervals (CI) for progression to Hurley stage III.

Using univariable logistic regression analysis with correction for multiple testing, several clinical laboratory values were identified as predictors of advanced HS (*q* < 0.05; Figure 3; Supplementary File 2). Significant positive associations were observed for neutrophil-related indices, including WBC (OR, 1.250; 95% CI, 1.079–1.447; *q* = 0.017), ANC (OR, 1.264; 95% CI, 1.080–1.479; *q* = 0.017), and NEUT% (OR, 1.071; 95% CI, 1.018–1.126; *q* = 0.028). In addition, LY% (OR, 0.926; 95% CI, 0.875–0.979; *q*=0.028) and red blood cell indices (hemoglobin, MCV, MCH, MCHC, and RDW) also showed significant associations with disease severity.

Biochemical markers of liver function were likewise independently associated with progression to Hurley stage III. Compared with patients in Hurley stages I–II, those with Hurley stage III had higher TP (OR, 1.171; 95% CI, 1.087–1.262; *q* = 0.0001), but lower albumin (OR, 0.880; 95% CI, 0.794–0.975; *q* = 0.033), ALT (OR, 0.980; 95% CI, 0.965–0.996; *q*=0.028), AST (OR, 0.953; 95% CI, 0.918–0.989; *q*=0.022), and TB (OR, 0.896; 95% CI, 0.824–0.974; *q*=0.028).

Variables with *q* < 0.05 in the univariable analysis were then entered into a multivariable logistic regression model. TP (OR, 1.240; 95% CI, 1.101–1.397; *q* = 0.010) remained an independent risk factor for progression to Hurley stage III (Supplementary File 3).

### 3.5 Receiver operating characteristic (ROC) analysis

**Figure 4:**
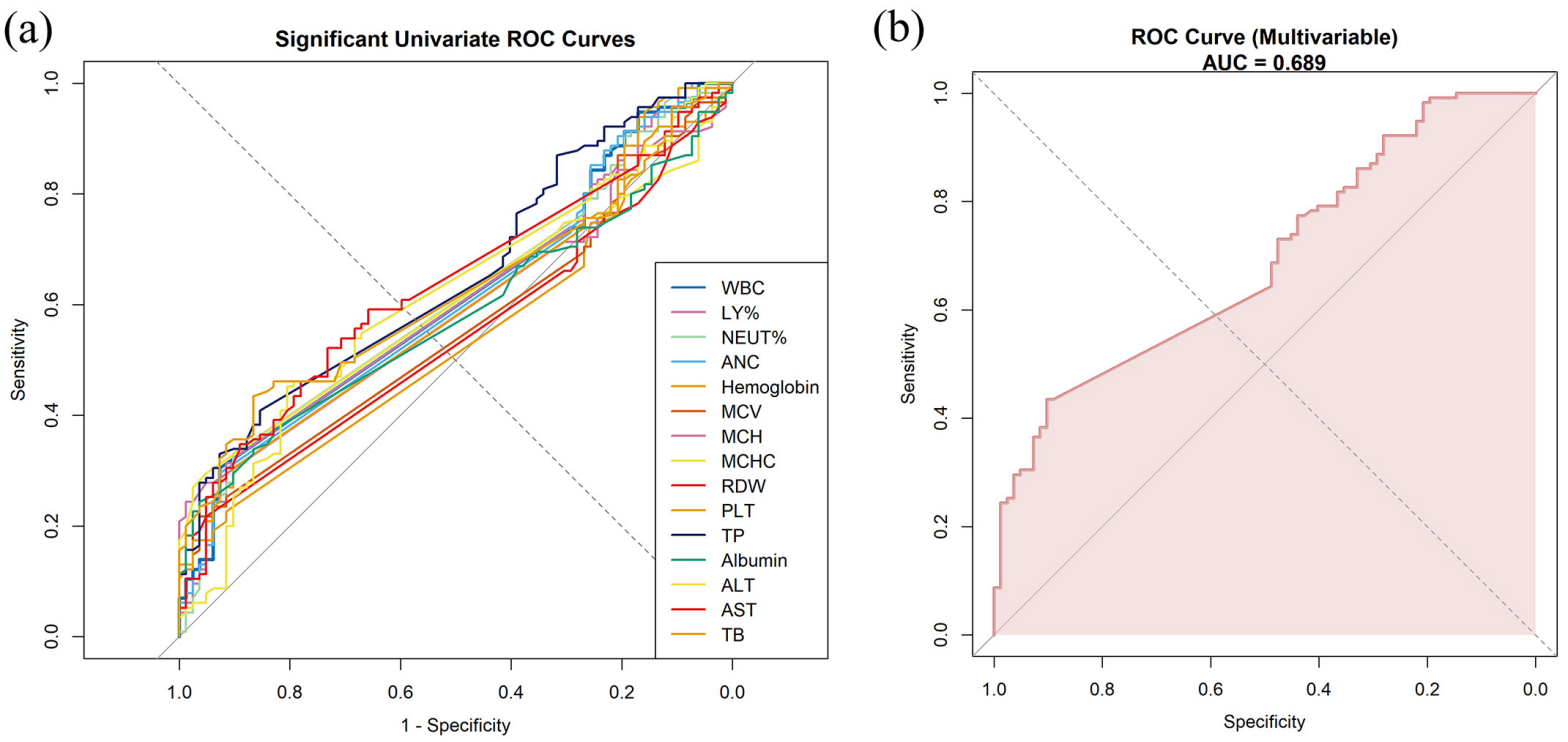
Receiver operating characteristic (ROC) analysis demonstrating the predictive performance of clinical and laboratory markers for progression to Hurley stage III, with the area under the curve (AUC). **(a)** ROC curves based on univariable logistic regression for significant markers. **(b)** ROC curve based on a multivariable logistic regression model integrating independent predictors (WBC, NEUT%, ANC, TP, and albumin).

ROC analysis was performed to evaluate the ability of clinical and laboratory markers to discriminate progression to Hurley stage III. Neutrophil-related indices (WBC, NEUT%, and ANC) showed modest predictive ability, with AUCs of 0.610, 0.608, and 0.605, respectively. Liver function markers showed slightly better performance, with AUCs of 0.652 for TP, 0.632 for AST, 0.619 for TB, and 0.612 for ALT (Figure 4a and Supplementary File 4).

Building on these findings, a multivariable logistic regression model incorporating key predictors identified in the univariable analysis demonstrated moderate discrimination, with an AUC of 0.689 (95% CI: 0.617–0.761). At the optimal cut-off (0.621), the model achieved high specificity (0.902) and a high positive predictive value (0.862), supporting its reliability in identifying patients at risk of progression to Hurley stage III (Figure 4b and Supplementary File 5).

## 4 Discussion

A striking male predominance was observed in this study, contrasting with most Western cohorts but consistent with Asian studies and our prior nationwide findings (male-to-female ratio, 4.7:1) (20–23). Although women represented a smaller proportion, they experienced a shorter diagnostic delay (*p* = 0.011) than males. This may reflect limited recognition of mild HS in community hospitals, where women with small nodules often receive local treatment rather than being referred, resulting in a referral bias toward more severe cases in our center. Sociocultural factors also play an important role. Women may be reluctant to seek medical care for lesions in intimate areas because of cultural modesty and disease-related stigma, especially when consultations involve male physicians, whereas men generally show less hesitation (24). Moreover, lower estradiol levels in Chinese women may attenuate hormonal sensitivity (25). In contrast, elevated androgen levels in men can activate the NLRP3 inflammasome and Th17 responses, enhancing neutrophil recruitment in HS lesions (26). Asian male patients are also more likely to carry γ-secretase variants that impair Notch signaling and promote keratinocyte dysregulation (27). Besides, lifestyle factors appear to amplify these differences. In Western cohorts, the overall smoking prevalence has been reported to reach up to 90%, whereas our cohort showed a lower rate of 56.5%, with a notable predilection for males (*p* < 0.001) (28). Finally, although obesity is more common in women in Western populations, male patients in our cohort had higher BMI than females (29). This reversal weakens the obesity-related risk for women in Chinese populations, and underscores smoking as the key lifestyle factor behind the male predominance in HS.

Chinese patients with HS showed milder metabolic abnormalities than Western populations (27). In our cohort, obesity, low HDL-c, and hyperglycemia were the most prevalent abnormalities, whereas hypertriglyceridemia, elevated TC, and LDL-c were less frequent. By contrast, in a German population study, central obesity (65.0%), reduced HDL-c levels (50.0%), and hypertriglyceridemia (38.8%) were more common, although the prevalence of hyperglycemia was slightly lower (26.3%) (30). An American study further reported more pronounced metabolic disturbances, with 87.6% of HS patients being obese, 43.8% having hypertriglyceridemia, 46.3% exhibiting low HDL-c, and 61.8% showing glucose intolerance (31). Within our cohort, further analysis showed that low HDL-c did not completely overlap with obesity, suggesting that it may represent an independent metabolic feature of HS. This is consistent with the findings of Hernández et al., (32) who reported that decreased HDL-c in HS cannot be attributed solely to obesity but may result from inflammation-driven mechanisms, such as MMP8-mediated degradation of ApoA1. Moreover, triglyceride levels were significantly higher in obese patients, yet neither TG nor HDL-c levels were independently associated with Hurley stage (31, 33). Taken together, these results suggest that metabolic abnormalities are more likely background comorbidities rather than direct drivers of HS severity, although they may still contribute to systemic inflammatory burden (34).

Neutrophils emerged as key mediators of inflammation in HS. Correlation analyses revealed that neutrophil-related indices, including WBC, ANC, and NEUT%, increased with disease severity, while LY% decreased. Logistic regression and ROC analyses further supported their predictive capacity in identifying patients at risk of progression to Hurley stage III. These results are consistent with previous studies, showing that the neutrophil-to-lymphocyte ratio (NLR) reflects the balance between innate and adaptive immunity. An elevated NLR indicates increased neutrophils with relative lymphopenia, a pattern typical of chronic inflammation (35–37). Mechanistically, sustained neutrophil activation releases proteolytic enzymes and inflammatory mediators such as MMP-9 and IL-1β, leading to local tissue damage. Activated neutrophils also form neutrophil extracellular traps (NETs), which amplify systemic inflammation and contribute to thrombosis, thereby perpetuating the inflammatory cycle. Persistent neutrophil activation promotes abscess formation, tunnel development, and chronic tissue remodeling, ultimately driving disease progression (36, 38–40). Therapeutically, targeting neutrophils is emerging as a promising strategy in HS. IL-1 inhibitors such as bermekimab and lutikizumab aim to suppress upstream inflammatory pathways, while interventions targeting chemokines, leukotriene B4, or G-CSF signaling seek to limit neutrophil recruitment (1). Historical anti-neutrophilic drugs, including dapsone, colchicine, and tetracyclines, have also shown variable benefit (41). Collectively, these findings emphasize neutrophil-mediated inflammation as a central pathogenic pathway and a promising therapeutic target in HS.

Although total protein (TP) is not a conventional inflammatory marker, it showed notable discriminatory capacity in our cohort. TP levels increased with HS severity, suggesting its potential as a helpful serological indicator. Albumin, in contrast, was negatively associated with Hurley stage, indicating that nutritional decline may accompany disease progression. Conversely, CRP and fibrinogen were positively associated with disease severity, supporting the interpretation that TP elevation largely reflects the accumulation of acute-phase proteins and thus captures the chronic inflammatory burden of HS (42). Previous evidence reported that elevated acute-phase proteins occur in both acute and chronic inflammation and are accompanied by reduced hepatic albumin synthesis (42). In addition, Jennifer Panara et al. (43) reported that the protein gap (total protein minus albumin) correlated strongly with HS severity, with each 1 g/dl increase associated with a 2.24-fold rise in IHS4 score. Accordingly, we speculate that elevated TP with normal albumin may reflect an inflammatory HS subtype (typically Hurley stage II) that mainly responds to anti-inflammatory therapy. In contrast, elevated TP with reduced albumin may indicate a subtype characterized by both inflammation and nutritional depletion (commonly Hurley stage III), which may benefit from combined anti-inflammatory and nutritional interventions.

Several limitations should be acknowledged. First, the cross-sectional design limits the ability to establish causality. Second, as a single-center study, the generalizability of our findings may be restricted. Third, the predominance of referred patients in our cohort introduces potential referral bias, which may affect the broader applicability of our results. To address this limitation, multi-center collaborations with larger sample sizes are warranted. Additionally, disease severity could not be uniformly assessed using the International Hidradenitis Suppurativa Severity Score System (IHS4) because standardized lesion counts were not consistently available in historical records, which may reduce the precision of severity classification.

In conclusion, Chinese patients with HS exhibited a pronounced male predominance and relatively mild metabolic abnormalities. Neutrophil-related indices and TP, especially when TP was interpreted together with albumin, were associated with progression to Hurley stage III.

## Data Availability

The original contributions presented in the study are included in the article/Supplementary material, further inquiries can be directed to the corresponding authors.

## Supplementary material

Supplementary File 1 Correlation analyses between clinical and laboratory parameters.

Supplementary File 2 Univariate logistic regression results.

Supplementary File 3 Multivariate logistic regression results.

Supplementary File 4 ROC analyses based on univariate models.

Supplementary File 5 ROC analyses based on multivariate models.
